# SARS-CoV-2 viremia and COVID-19 mortality: A prospective observational study

**DOI:** 10.1371/journal.pone.0281052

**Published:** 2023-04-28

**Authors:** Andrea Giacomelli, Elena Righini, Valeria Micheli, Pietro Pinoli, Anna Bernasconi, Alberto Rizzo, Letizia Oreni, Anna Lisa Ridolfo, Spinello Antinori, Stefano Ceri, Giuliano Rizzardini

**Affiliations:** 1 Dipartimento di Malattie Infettive, ASST-FBF-Sacco, Milano, Italia; 2 Dipartimento di Elettronica, Informazione e Bioingegneria (DEIB), Politecnico di Milano, Milano, Italia; 3 Laboratory of Clinical Microbiology, Virology and Bioemergencies, ASST-FBF-Sacco, Milan, Italy; 4 Dipartimento di Scienze Biomediche e Cliniche Luigi Sacco, Università degli Studi di Milano, Milano, Italia; Non-Communicable Diseases Research Center, Endocrinology and Metabolism Research Institute, Tehran University of Medical Sciences, IRAN, ISLAMIC REPUBLIC OF

## Abstract

**Background:**

SARS-CoV-2 viremia has been found to be a potential prognostic factor in patients hospitalized for COVID-19.

**Objective:**

We aimed to assess the association between SARS-CoV-2 viremia and mortality in COVID-19 hospitalized patients during different epidemic periods.

**Methods:**

A prospective COVID-19 registry was queried to extract all COVID-19 patients with an available SARS-CoV-2 viremia performed at hospital admission between March 2020 and January 2022. SARS-CoV-2 viremia was assessed by means of GeneFinderTM COVID-19 Plus RealAmp Kit assay and SARS-CoV-2 ELITe MGB^®^ Kit using <45 cycle threshold to define positivity. Uni and multivariable logistic regression model were built to assess the association between SARS-CoV-2 positive viremia and death.

**Results:**

Four hundred and forty-five out of 2,822 COVID-19 patients had an available SARS-CoV-2 viremia, prevalently males (64.9%) with a median age of 65 years (IQR 55-75). Patients with a positive SARS-CoV-2 viremia (86/445; 19.3%) more frequently presented with a severe or critical disease (67.4% vs 57.1%) when compared to those with a negative SARS-CoV-2 viremia. Deceased subjects (88/445; 19.8%) were older [75 (IQR 68-82) vs 63 (IQR 54-72)] and showed more frequently a detectable SARS-CoV-2 viremia at admission (60.2% vs 22.7%) when compared to survivors. In univariable analysis a positive SARS-CoV-2 viremia was associated with a higher odd of death [OR 5.16 (95% CI 3.15-8.45)] which was confirmed in the multivariable analysis adjusted for age, biological sex and, disease severity [AOR 6.48 (95% CI 4.05-10.45)]. The association between positive SARS-CoV-2 viremia and death was consistent in the period 1 February 2021–31 January 2022 [AOR 5.86 (95% CI 3.43-10.16)] and in subgroup analysis according to disease severity: mild/moderate [AOR 6.45 (95% CI 2.84-15.17)] and severe/critical COVID-19 patients [AOR 6.98 (95% CI 3.68-13.66)].

**Conclusions:**

SARS-CoV-2 viremia resulted associated to COVID-19 mortality and should be considered in the initial assessment of COVID-19 hospitalized patients.

## Introduction

Severe acute respiratory syndrome coronavirus 2 (SARS-CoV-2) infection determines coronavirus disease 2019 (COVID-19) ranging from asymptomatic/paucisymptomatic diseases to severe and critical ones requiring hospital admission and oxygen support [1]. The SARS-CoV-2 pandemic evolved starting with a rapid and unexpected surge of critically ill patients firstly in China [1] and then in Lombardy which was the first epicentre of the European epidemic in late February 2020 [2]. The unprecedented number of severe and critically ill respiratory patients claimed the identification of factors associated with severe courses and fatal outcomes of COVID-19 patients to be performed at the time of hospital admission [3]. Since the beginning of the pandemic age, gender and obesity appeared to be major determinants of COVID-19 severity and mortality [4, 5]. Apart from these demographic and clinical characteristics, SARS-CoV-2, viremia has been found to be a potential marker of disease severity and a prognostic factor in patients hospitalized for COVID-19 [6, 7]. This finding was not unexpected considering that it has been demonstrated in the previous SARS epidemic that a high serum viral load and detection of virus at multiple sites were predictive of adverse clinical outcomes [8]. It has been speculated that an uncontrolled viremia could be the possible cause of severe disease or even the driver of the inflammation that characterizes severe COVID-19 [9, 10]. Most of the studies were carried out during the first and second epidemic waves of COVID-19 characterized by a suboptimal management of patients in terms of respiratory support, overloaded health care facilities, the lack of specific treatments and the absence of an effective vaccine [11, 12]. In addition, the majority of the studies mainly focused on critically ill hospitalized subjects already with an advanced disease with an unfavo urable prognosis [10, 13, 14]. Thus, little is known about the prognostic value of SARS-CoV-2 viremia in a different epidemiological scenario characterized by an improved patient care and the availability of vaccines. In addition, it is unclear if SARS-CoV-2 viremia is associated to worse outcome also in subjects with mild to moderate COVID-19.

The aim of our study was to test the association between SARS-CoV-2 viremia performed at the time of hospital admission and mortality in COVID-19 hospitalized patients during different epidemic periods.

## Materials and methods

### Study design and setting

This prospective cohort study enrolled all of the adult COVID-19 patients admitted to the Department of Infectious Diseases and the Intensive Care Unit (ICU) of Luigi Sacco Hospital. The characteristics of the hospital and the rapid reorganization necessity in order to respond to the pandemic have been previously described [4, 15–18]. For the purposes of the present analysis, the Luigi Sacco COVID-19 hospital-based registry was queried to extract all COVID-19 subjects with an available SARS-CoV-2 viremia performed at hospital admission.

### Participants

The analysis considered all of the adult (≥18 years of age) COVID-19 patients admitted to the hospital’s Department of Infectious Diseases and ICU between 1st March 2020 (the day in which the SARS-CoV-2 viremia test was implemented at our center) and 31st January 2022, who were observed until the time of death or discharged alive, whichever came first. The diagnosis of COVID-19 was confirmed by a positive real-time reverse-transcription polymerase chain reaction on a nasopharyngeal swab. Subjects with a SARS-CoV-2 viremia determination were used to test the study hypothesis, whereas concurrent COVID-19 hospitalized subjects were used to assess potential bias in SARS-CoV-2 viremia requests during the study period.

### Data collection

The characteristics of our registry data management have been described elsewhere [4, 15–18]. In brief, the data were extracted from the patients’ clinical charts on a daily basis and stored in an ad hoc database. The collected data were the patients’ date and place of birth, and biological sex; the date of admission, and the time between symptom onset and hospital admission; comorbidities (including diabetes, lung diseases, heart diseases, renal diseases, immune system diseases, liver diseases, and obesity); the burden of comorbidities assessed by means of Charlson Comorbidity Index (CCI); disease severity upon hospital admission (defined as mild, moderate, severe or critical in accordance with the WHO guidelines for the management of COVID-19) [19]; the type of supportive oxygen therapy upon hospital admission and/or during the hospital stay; the drugs used to treat COVID-19 (which included hydroxychloroquine, lopinavir/ritonavir, remdesivir, tocilizumab and other immunomodulators, heparin, and steroids); the number of COVID-19 vaccine shot received (none, 1, 2 and 3) and the hospitalization outcome (death and discharged alive).

The vital status of the patients who were transferred to other facilities was ascertained by means of telephone calls and, when appropriate, during an on-site examination by a member of our dedicated post-COVID-19 outpatient service.

For the purposes of this study, the patients were arbitrary divided into four groups on the basis of the time of their hospital admission. The four consecutive periods were characterized by surges in COVID-19 cases and hospitalizations in Italy: 1 March–31 May 2020 (first wave); 1 August 2020–31 January 2021 (second wave); 1 February–30 June 2021 (third wave); and 1 July 2021–31 January 2022 (fourth wave) [20, 21].

### Laboratory procedures

In order to detect SARS-CoV-2 in the bloodstream, blood specimens were collected in BD Vacutainer^®^ K2EDTA tube to obtain plasma. Samples collected from March 2020 to February 2021 were tested for SARS-CoV-2 RNA by means of GeneFinderTM COVID-19 Plus RealAmp Kit assay (targets: E, N and RdRP genes) (ELITechGroup Italy). Starting from March 2021, samples were tested using SARS-CoV-2 ELITe MGB^®^ Kit (targets: RdRP and ORF8 genes) (ELITechGroup Italy). The change of SARS-CoV-2 assay by the manufacturer represents an optimization of SARS-CoV-2 detection through a more appropriate target genes choice. The detection of one target is the minimum condition to define a positive result and the cut-off of the assay is <45 cycle threshold (Ct).

### Outcomes

The main outcome of interest was death.

### Data analysis

The descriptive statistics show proportions for categorical variables, and median values with their interquartile range (IQR) for continuous variables.

The baseline demographic and clinico-epidemiological characteristics of the patients according to the COVID-19 outcome (death vs discharged alive) were compared using the *χ*^2^ or, when necessary, Fisher’s exact test in the case of categorical variables, and Wilcoxon’s rank-sum test in the case of continuous variables. The characteristics of patients were also compared based on the SARS-CoV-2 viremia result (positive vs negative) and on the availability of a SARS-CoV-2 viremia test at hospital admission (yes vs not).

Univariable Logistic regression models were used to investigate the relationship between the SARS-CoV-2 viremia and the outcome of interest (death). Associations were estimated using odds ratios (ORs) with their 95% confidence intervals (CIs). Multivariable Logistic regression models were used to adjust the ORs for potential confounders including age, biological sex, and disease severity upon admission, and the results were expressed as adjusted odds ratios (AORs) with their 95% CIs.

We hypothesized that the evolution of COVID-19 management and the changing epidemiological situation could have affected the estimates of association between SARS-CoV-2 viremia and death. Consequently, a sub-analysis of the risk of death by means of univariable and multivariable Logistic regression analysis was carried out restricted to subjects hospitalized from 1 February 2021–31 January 2022 (corresponding to the third and fourth epidemic waves’ period) [20, 21].

To assess the association between SARS-CoV-2 positive viremia and death in patients with different disease severity a sub analysis was carry out restricted to mild/moderate and severe/critical COVID-19 patients overall and restricted to the 3 and 4 epidemic waves.

### Ethical approval and consent to participate

The study was approved by our Ethics Committee [Comitato Etico Interaziendale Area 1, Milan, Italy (Protocol No. 16088)]. All patients signed a written informed consent except those on mechanical ventilation upon admission from whom was waived according to the Ethics Committee (Comitato Etico Interaziendale Area 1, Milan, Italy). All methods were carried out in accordance with the Helsinki declaration and the manuscript follows the STROBE checklist for observational studies. If the patient was able to make informed decisions about their own medical care and participation into the study the informed consent was directly obtained. Otherwise, if the patient was not able to provide an informed decision regarding his/her/him own medical care and participation into the study the informed consent was obtained by his/her/him legal guardian.

## Results

### Characteristics of the study population

During the study period 2,822 COVID-19 patients were enrolled in the Luigi Sacco COVID-19 hospital registry; out of these, 520 (18.4%) died (S1 Table). The overtime number of hospitalizations per weeks is depicted in S1 Fig. Four hundred and forty-five (18.5%) COVID-19 patients had an available SARS-CoV-2 viremia during the study period of whom 88 (19.8%) died. Patients tested for SARS-CoV-2 viremia more frequently presented with a severe or critical COVID-19 (59.1% vs 48.5%; p<0.001), showed a longer median time from symptoms onset to hospital admission [8 days (IQR 5–11) vs 7 days (IQR 4–10); p = 0.004] and a higher proportion of patients was tested during the 3rd epidemic wave (50.6% vs 20.5%; p<0.001) when compared to those without a SARS-CoV-2 viremia determination.

### Characteristics of patients with an available SARS-CoV-2 viremia

**Fig 1:**
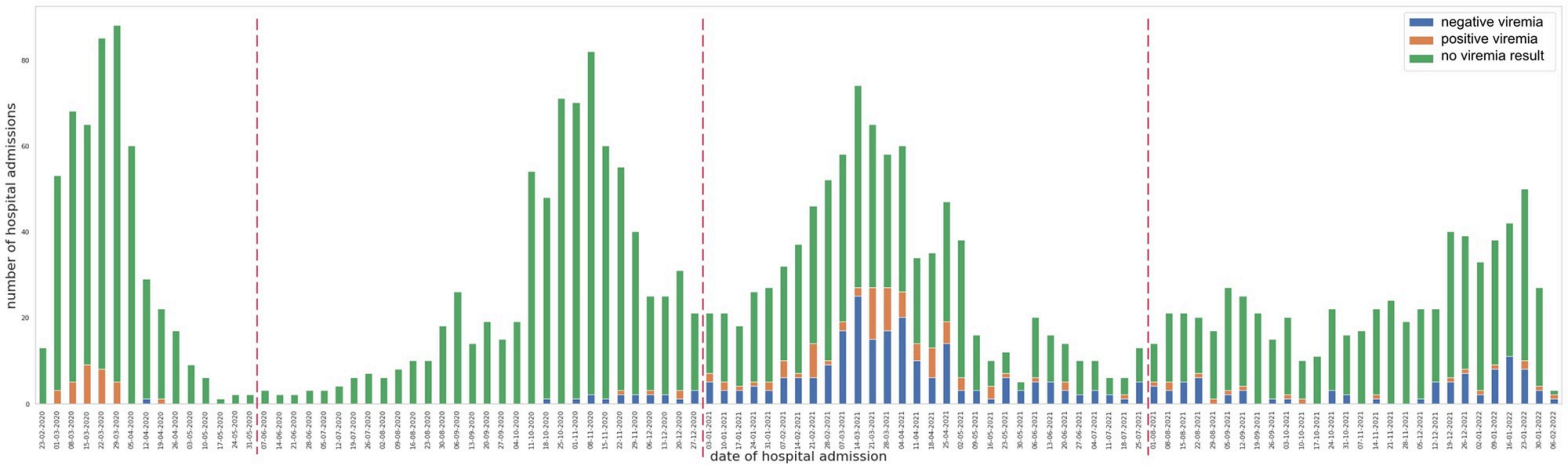
Stacked bar chart. Number of hospital admissions with positive, negative viremia and with no viremia result.

The overtime number per week of COVID-19 patients tested for SARS-CoV-2 viremia according to being tested positive or negative is depicted in Fig 1. No patient was found to be positive for SARS-CoV-2 viremia above 38 Ct. The characteristics of subjects with an available SARS-CoV-2 viremia according to being COVID-19 survivors or not are reported in Table 1. They were prevalently males (64.9%) with a median age of 65 years (IQR 55–75). Deceased subjects were older [75 (IQR 68–82) vs 63 (IQR 54–72); p<0.001], presented more frequently with a severe or critical disease at hospital admission (70.5% vs 56.3%; p = 0.0157), had a higher median CCI [4 (IQR 3–6) vs 2 (1–4); p<0.001] and showed more frequently a positive SARS-CoV-2 viremia at admission (60.2% vs 22.7%; p<0.001) when compared to survivors. Characteristics of subjects according to the SARS-CoV-2 viremia result are reported in S2 Table. Patients with a positive SARS-CoV-2 viremia were overrepresented in the first epidemic wave (35.6% vs 0.3%; p<0.001), more frequently presented with a severe or critical disease at hospital admission (67.4% vs 57.1%; p = 0.088) and more frequently died (34.9% vs 16.2%; p<0.001) when compared to those with a negative SARS-CoV-2 viremia at hospital admission.

**Table 1:** Characteristics of the study population according to being alive or death after hospitalization for COVID-19.

Characteristics	Overall	Alive	Death
	445 (100%)	357 (80.2%)	88 (19.8%)
**Male biological sex, n (%)**	289 (64.9)	229 (64.1)	60 (68.2)
**Age, years**			
median (IQR)	65 (55–75)	63 (54–72)	75 (68–82)
>75 years, n (%)	120 (27)	72 (20)	48 (54)
**CCI, median (IQR)**	2.5 (1–4)	2 (1–4)	4 (3–6)
**SARS-CoV-2 pandemic wave, n (%)**			
1	58 (13)	47 (13.2)	11 (12.5)
2	21 (4.7)	15 (4.2)	6 (6.8)
3	255 (57.3)	200 (56)	55 (62.5)
4	111 (24.9)	95 (26.6)	16 (18.2)
**Days from symptoms onset to Hospital admission, median (IQR)**	8 (5–11)	8 (5–11)	7 (4–10)
**Disease severity at hospital admission, n (%)**			
Mild/moderate	182 (40.9)	156 (43.7)	26 (29.5)
Severe/critical	263 (59.1)	201 (56.3)	62 (70.5)
**Doses of COVID-19 Vaccine, n (%)**			
0	370 (83.1)	297 (83.2)	73 (83)
1	37 (8.3)	21 (5.9)	5 (5.7)
2	29 (6.5)	32 (9)	8 (9.1)
3	9 (2)	7 (2)	2 (2.3)
**Positive SARS-CoV-2 viremia at admission, n (%)**	134 (30.1)	81 (22.7)	53 (60.2)

List of abbreviations: n, number; IQR, Inter Quartile Range; CCI, Charlson comorbidity index.

### Characteristics of patients admitted during the 3^rd^ and 4^th^ epidemic waves

During the 3rd and 4th epidemic waves 1,346 COVID-19 patients were hospitalized of whom 364 (27.1%) had an available viremia (S3 Table). Patients tested for SARS-CoV-2 viremia more frequently presented with a severe or critical COVID-19 (58.5% vs 43%; p<0.001), a higher proportion of patients was tested during the 3rd epidemic (70.1% vs 49.6%; p<0.001) and more frequently died (19.2% vs 12.1%; p<0.001) when compared to those without a SARS-CoV-2 viremia determination. Seventy (19.2%) out of 364 subjects died and d Deceased subjects were older [76 (68–83) vs 63 (54–72); p<0.001], presented more frequently with a severe or critical disease at hospital admission (71.4% vs 55.4%; p = 0.0153), had a higher median CCI [4 (IQR 3–6) vs 2 (1–4); p<0.001], a shorter median time from symptoms onset to hospital admission [7 days (IQR 4–11) vs 8 days (IQR 5–11); p<0.001] and showed more frequently a positive SARS-CoV-2 viremia at admission (54.3% vs 17.7%; p<0.001) when compared to survivors (S4 Table). Patients with a positive SARS-CoV-2 viremia more frequently died (38% vs 16.2%; p<0.001) when compared to those with a negative SARS-CoV-2 viremia at hospital admission (S5 Table).

### Factors associated with the risk of COVID-19-related deathUni and multivariable logistic regression analysis

In univariable analysis a positive SARS-CoV-2 viremia was associated with a higher odd s of death [OR 5.16 (95% CI 3.15–8.45)] such as age (per 1 years more) [OR 1.01 (95% CI 1.01–1.02)] and disease severity at hospital admission (severe/critical vs mild/moderate) [OR 1.85 (95% CI 1.12–3.06)] whereas being female was associated with lower odds of death [OR 0.57 (95% CI 0.35–0.94)] (Table 2). In multivariable analysis adjusted for age, biological sex and, disease severity a positive SARS-CoV-2 viremia resulted associated with higher odds of death [AOR 6.48 (95% CI 4.05–10.45)].

**Table 2:** Logistic regression analysis of factors associated with death.

Characteristics	OR	95%CI	aOR	95%CI
**Females** ***vs*** **Males**	0.57	0.35–0.94	0.63	0.38–1.03
**Age** (per 1 year more)	1.01	1.01–1.02	1.09	1.06–1.11
**CCI** (per one point more)	0.99	0.98–1.02	–	–
**SARS-CoV-2 epidemic waves**				
1 *vs* 2	1.71	0.54–5.41	–	–
1 *vs* 3	1.18	0.57–2.42	–	–
1 *vs* 4	0.72	0.31–1.67	–	–
**Days from symptoms onset** (per one day more)	1.00	1.00–1.01	–	–
**Disease severity** (Mild/moderate vs severe/critical)	1.85	1.12–3.06	1.55	0.95–2.54
**Doses of vaccine**				
0 *vs* 1	0.97	0.35–2.66	–	–
0 *vs* 2	1.02	0.45–2.30	–	–
0 *vs* 3	1.16	0.24–5.71	–	–
**Positive SARS-CoV-2 viremia** ***vs*** **negative**	5.16	3.15–8.45	6.48	4.05–10.55

List of abbreviations: OR, odds ratio; CI, confidence interval; aOR, adjusted odds ratio; CCI, Charlson comorbidity index.

In univariable analysis restricted to the 3rd and 4th epidemic waves a positive SARS-CoV-2 viremia was associated with a higher odd of death [OR 5.69 (95% CI 3.58–9.11)] which was confirmed in the multivariable analysis adjusted for age, biological sex and disease severity [AOR 5.86 (95% CI 3.43–10.16)] (Table 3).

**Table 3:** Logistic regression analysis of factors associated with death restricted to the 3rd and 4th epidemic period.

Characteristics	OR	95%CI	aOR	95%CI
**Females** ***vs*** **Males**	0.94	0.59–147	0.72	0.42–1.22
**Age** (per 1 year more)	1.08	1.06–1.10	1.09	1.06–1.12
**CCI** (per one point more)	1.39	1.26–1.54	–	–
**Days from symptoms onset** (per one day more)	0.99	0.95–1.02	–	–
**Doses of vaccine**				
0 *vs* 1	0.65	0.26–1.41	–	–
0 *vs* 2	1.59	0.74–3.22	–	–
0 *vs* 3	1.19	0.25–4.10	–	–
**Positive SARS-CoV-2 viremia** ***vs*** **negative**	5.69	3.58–9.11	5.86	3.43–10.16

List of abbreviations: OR, odds ratio; CI, confidence interval; aOR, adjusted odds ratio; CCI, Charlson comorbidity index.

In univariable analysis restricted to mild/moderate COVID-19 patients a positive SARS-CoV-2 viremia was associated with a higher odd of death [OR 4.57 (95% CI 2.19–9.55)] which was confirmed in the multivariable analysis adjusted for age and, biological sex [AOR 6.45 (95% CI 2.84–15.17)]. After restricting the analysis to the 3rd and 4th epidemic waves in mild/moderate COVID-19 patients a positive SARS-CoV-2 viremia was associated with a higher odd of death [OR 3.89 (95% CI 1.63–9.06)] which was confirmed in the multivariable analysis adjusted for age and, biological sex [AOR 4.98 (95% CI 1.89–13.29)].

In univariable analysis restricted to severe/critical COVID-19 patients a positive SARS-CoV-2 viremia was associated with a higher odd of death [OR 5.07 (95% CI 3.07–8.53)] which was confirmed in the multivariable analysis adjusted for age and, biological sex [AOR 6.53 (95% CI 3.68–11.93)]. After restricting the analysis to the 3rd and 4th epidemic waves in severe/critical COVID-19 patients a positive SARS-CoV-2 viremia was associated with a higher odd of death [OR 6.19 (95% CI 3.51–11.11)] which was confirmed in the multivariable analysis adjusted for age and, biological sex [AOR 6.98 (95% CI 3.68–13.66)].

## Discussion

In our study we found that a positive SARS-CoV-2 viremia is associated with death in COVID-19 hospitalized patients. The estimates of association were consistent in more recent epidemic periods, suggesting that SARS-CoV-2 viremia could still be an important prognostic biomarker to be performed at hospital admission.

In our study we found that 19.3% COVID-19 patients tested for SARS-CoV-2 viremia showed a positive result a value that is significantly lower when compared to the 50% reported in 2003 for SARS-CoV [22, 23]. It is worth mentioning that the proportion of positive viremia observed in our study was different in the 1st and 2nd epidemic periods when compared to the 3rd and 4th ones showing a significant lower proportion of positive subjects in the latter (44.5% vs 13.7%). Our finding of a high proportion of viremic patients during the 1st and 2nd epidemic waves is in line with a study conducted in the early epidemic period in China [6], another retrospective study conducted by Rodríguez Serrano et al. in the early days of the epidemic in Spain [24] and another study conducted in the US [25] showing a 41%, 50–60% and 38% of viremic hospitalized COVID-19 patients, respectively. On the contrary, it was higher when compared to the 29% (121/417) reported by Hagman et al. [26] in a study conducted in Sweden covering the period April-September 2020 and two other studies, one conducted in Spain between November 2020 and January 2021 in which 14 out of 57 (24.6%) subjects showed a positive viremia at hospital admission [27] and another study conducted in Japan between April 2020 and September 2020 in which a positive RNAemia was detected in 19.6% (11/56) patients on hospital admission [28]. In a meta-analysis [29] covering the published literature between December 2019 and December 2020 including 21 studies involving 2,181 COVID-19 patients SARS-CoV-2 RNAemia in COVID-19 patients varied from 9.4% to 74.1%, with a pooled estimate of 34% (95%CI 26%-43%). The between-study variability relies on the different case mix and wide clinical spectrum of COVID-19 disease. In fact, as observed in our study, a positive viremia was more frequently found in patients with a more severe disease. In addition, most of the conducted studies were retrospective in nature, with different times of sampling, covering a different time span, different epidemic scenario, and different PCR techniques [29]. However, little is known regarding the rates of positive viremia in subjects hospitalized after the 1st and 2nd epidemic periods. Our study fills this gap by showing a significant lower proportion of viremic subjects 13.7% in the 3rd and 4th epidemic period. This seems not to be related to the selection of patients to be tested with a less severe disease or with a lower proportion of worse outcome. On the contrary, it could be speculated that different viral dynamics between the study period occurred. Nevertheless, whether they are related to different treatments, different circulating variants or other unmeasured confounders or patient’s selection bias could not be definitely determined.

In our study we found that SARS-CoV-2 viremia was associated with a higher odd s of death consistently with the available literature again mainly produced on data collected during the early days of the pandemic and on subjects with a more severe course of the disease [13]. These findings are in line with SARS-CoV and Middle East respiratory syndrome (MERS), in which the detection of the virus in plasma correlated with adverse clinical outcome [30, 31]. In addition, in a study conducted during the first epidemic wave the frequency of SARS-CoV-2 viremia has been found to be higher in critically ill patients when compared to non -critical hospitalized ones and to outpatients (78% vs 27% vs 2%, respectively) [10]. In the above -mentioned meta-analysis [29], SARS-CoV-2 RNAemia was found to be associated not only with COVID-19 severity [OR 5.43 (95%CI 3.46–8.53)] but also with death OR [11.07 (95%CI 5.60–21.88)]. This association was found mainly in studies conducted on critically ill COVID-19 patients [32, 33]. In a Swedish cohort the SARS-CoV-2 RNA positivity resulted associated with a n hazard ratios for critical disease and all-cause mortality of 7.2 (95%CI 3.0–17) and 8.6 (95%CI 2.4–30), respectively [7]. These estimates are consistent with what has been observed in our cohort with 6.48 higher odds of death in COVID-19 patients with a detectable SARS-CoV-2 viremia in plasma, a finding that was to be found consistently also in the 3rd and 4th epidemic periods.

Some studies considered a viral load threshold to define viremia in critically ill patients and related this parameter to the risk of mortality. In a study conducted by Heinrich and colleagues blood RNA loads exceeding 2.51×103 SARS-CoV-2 RNA copies/mL were found to indicate a 50% probability of death [32]. In another study by Li et al., the lower limit of SARS-CoV-2 N gene quantification was 100 copies/mL and those subjects with detectable viral load were significantly more likely to have severe disease when compared to those without (82% vs 26%, respectively) [34]. In a study conducted on critically ill patients SARS-CoV-2 RNA copies above 1000/ml measured by PCR in plasma was defined as RNAemia and a higher mortality was observed in those patients with a positive viremia when compared to those without (35% vs 16%, respectively) [33]. According to the first evidence we considered as positive plasma samples with RT-PCR Ct value <38 and viremia was interpreted as a dichotomous variable. This could have direct implication in clinical practice with the performance of an easy-to-interpreted test at hospital admission able to identify patients predisposed to a worse clinical outcome, irrespectively from the well-known classical risk factors for COVID-19 mortality.

According to the manufacturer, the second release of the assay we introduced to assess viremia provided comparable specificity (100%) and an increased sensitivity (Limit of Detection—LoD: 100 versus 500 genome equivalents/mL compared to the first version). Considering these data, alongside the epidemiological and clinical characteristics of Omicron-dominated variant, it is unlikely that the lower frequency of positive viremia was related to the change of the assay. The reduced severity of Omicron infection and the consequent reduction in the number of patients hospitalized for COVID-19 are probably the most important driver of a reduced frequency of positive SARS-CoV-2 viremia.

Moreover, in a recent study conducted in Spain covering the period April 2021-May 2022 the authors found that a relevant viremia (defined as at least a two-fold increase in VL within ≤2 days or a VL>300 copies/mL) was associated with an increased risk of death [OR 13.5 (95%CI,6.3–28.7); p<0.0001] [27]. These findings are consistent with what we observed during the 3rd and 4th epidemic period and are supplemented by our analysis restricted to subjects with mild to moderate disease suggesting that SARS-CoV-2 viremia should be considered not only in patients presenting with a critical disease but also in those with a less severe clinical presentation.

### Study limitations and strength

Our study has several limitations. First, although the Luigi Sacco Hospital based registry is prospective in nature, our study was based on a retrospective search of subjects tested for SARS-CoV-2 viremia and thus prone to bias. Second, the study period covers a long time span in which significant improvements in COVID-19 management have occurred, thus exposing the study to maturation bias. Third, the request for SARS-CoV-2 viremia was at single physician discretion and thus prone to selection bias. FourthIn the end, two different SARS-CoV-2 viremia determination methods have been employed during the study period although it directly reflects the improvement of SARS-CoV-2 diagnostic technology observed during the study period with an improvement of the sensitivity. In the end, the unavailability in the dataset of Ct values for SARS-CoV-2 viremia does not allow us to test the correlation between the number of Ct at hospital admission (as a continuous variable) and the risk of death.

The main strength of the study relies on the large hospital-based cohort, which allowed us to assess the characteristics of subjects with and without viremia request enrolled in the registry during the same epidemic wave. This allowed us to check for potential selection and indication bias among COVID-19 patients address to be tested for SARS-CoV-2 viremia by their physicians. In addition, the long time span of our study allowed us to restrict the analysis excluding patients with the first and second epidemic waves, which were over-represented in available literature, and provide data on subsequent epidemic period characterized by an improved COVID-19 management, the circulation of different SARS-CoV-2 variants, and the implementation of COVID-19 vaccination. In the end, the dichotomous interpretation of SARS-CoV-2 viremia provides an easy to apply information in clinical practice.

## Conclusion

In our study we found that a positive SARS-CoV-2 viremia at hospital admission is associated with increased odds of death in subjects hospitalized for COVID-19. The estimates were consiste ntd after restricting the analysis only to subjects hospitalized in the third and fourth epidemic waves and thus with a less severe clinical presentation. Our study adds evidence to the notion that SARS-CoV-2 viremia could be used as an additional tool to assess the prognosis of COVID-19 hospitalized patients with SARS-CoV-2 infection other than *wild-type* virus and alfa variants. Whether SARS-CoV-2 viremia could drive to a different COVID-19 management in terms of treatment indication, i.e. with an antiviral agent, in the current epidemic situation is yet to be determined.

## Supporting information

S1 Fig Bar chart: Number of hospital admissions. (TIF)

S2 Fig Stacked bar chart: Number of death/dismissed patients. (TIF)

S3 Fig Stacked bar chart: Number of mild + moderate_severe + critical patients at hospital admission. (TIF)

S1 Table Characteristics of the study population according to being tested or not for SARS-CoV-2 viremia at hospital admission. (DOCX)

S2 Table Characteristics of the study population according to being tested positive or negative for SARS-CoV-2 viremia. (DOCX)

S3 Table Characteristics of the study population according to being tested or not for SARS-CoV-2 viremia at hospital admission restricted to the 3rd and 4th epidemic waves. (DOCX)

S4 Table Characteristics of the study population according to being alive or death after hospitalization for COVID-19 restricted to the 3rd and 4th epidemic waves. (DOCX)

S5 Table Characteristics of the study population according to being tested positive or negative for SARS-CoV-2 viremia restricted to the 3rd and 4th epidemic waves. (DOCX)

S1 Dataset (TSV)
