# Supplementary figures and images for: SARS-CoV-2 viremia and COVID-19 mortality: A prospective observational study
Source: PLoS One. 2023 Apr 28;18(4):e0281052. doi: 10.1371/journal.pone.0281052 (PMC10146509; doi:10.1371/journal.pone.0281052)

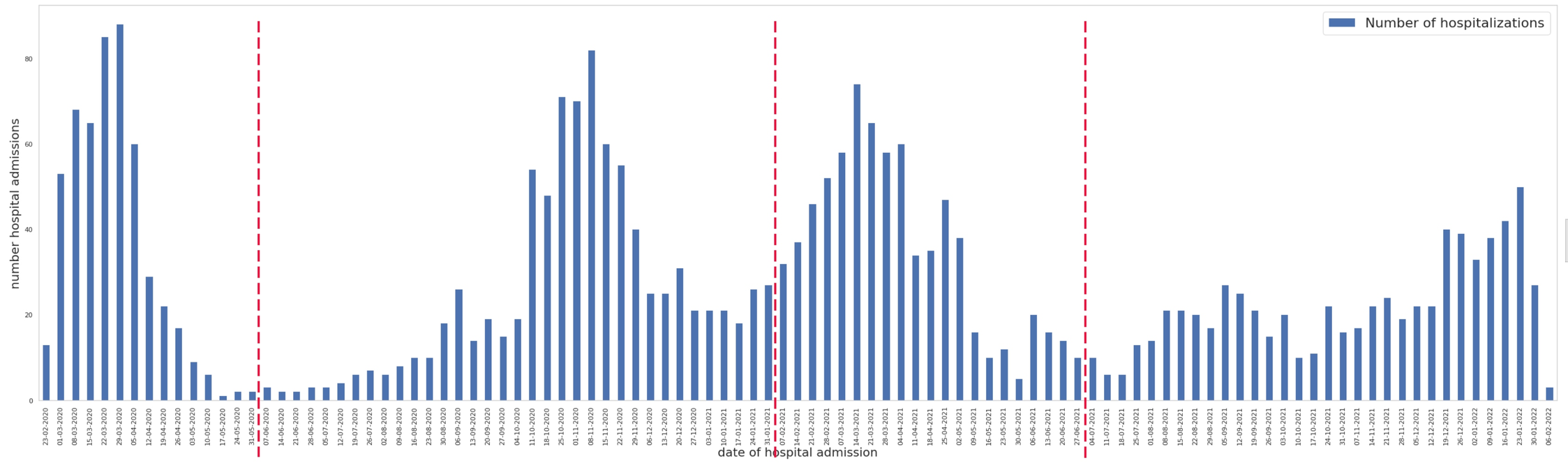

Supplement: S1 Fig — (TIF) [file pone.0281052.s001.tif]

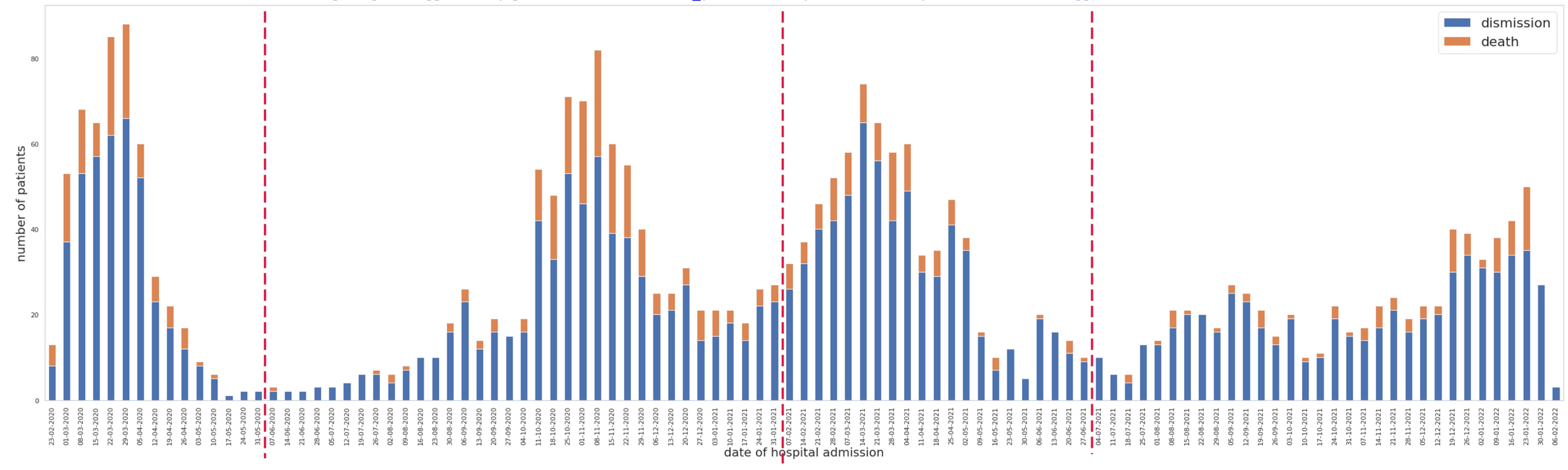

Supplement: S2 Fig — (TIF) [file pone.0281052.s002.tif]

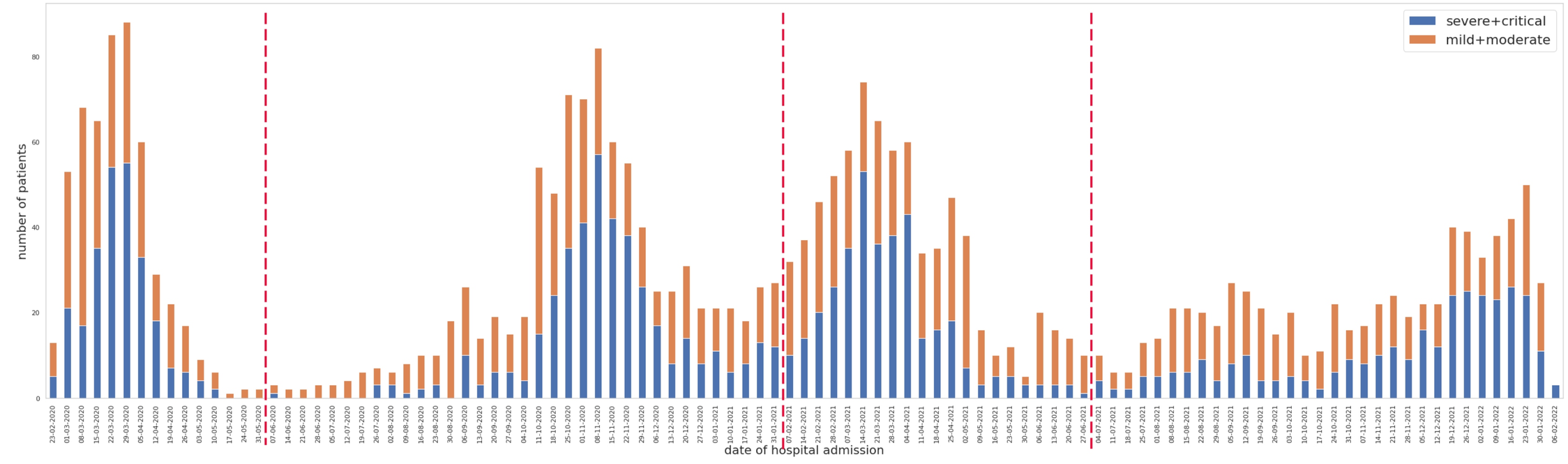

Supplement: S3 Fig — (TIF) [file pone.0281052.s003.tif]
