# Supplementary material for: SARS-CoV-2 viremia and COVID-19 mortality: A prospective observational study
Source: PLoS One. 2023 Apr 28;18(4):e0281052. doi: 10.1371/journal.pone.0281052 (PMC10146509; doi:10.1371/journal.pone.0281052)
Supplement: S1 Table — (DOCX) [file pone.0281052.s004.docx]

| Characteristic | Overall  2822  (100%) | Not tested for SARS-CoV-2 viremia  2377  (84.2%) | Tested for SARS-CoV-2 viremia  445  (15.8%) |
| --- | --- | --- | --- |
| Male biological sex, n (%) | 1763 (62.5) | 1474 (62) | 289 (64.9) |
| Age, years |  |  |  |
| median (IQR) | 66 (54-78) | 66 (54-78) | 65 (55-75) |
| >75 years, n (%) | 929 (32.9) | 809 (34) | 120 (27) |
| CCI, median (IQR) | 3 (1-5) | 3 (1-5) | 2.5 (1-4) |
| SARS-CoV-2 pandemic wave, n (%) |  |  |  |
| 1 | 520 (11.8) | 488 (20.5) | 32 (7.2) |
| 2 | 870 (30.8) | 823 (34.6) | 47 (10.6) |
| 3 | 742 (26.3) | 487 (20.5) | 255 (50.6) |
| 4 | 690 (24.5) | 579 (24.4) | 111 (24.9) |
| Days from symptoms onset to Hospital admission, median (IQR) | 7 (4-10) | 7 (4-10) | 8 (5-11) |
| Disease severity at hospital admission, n (%) |  |  |  |
| Mild/moderate | 1470 (52.1) | 1288 (54.2) | 182 (40.9) |
| Severe/critical | 1352 (47.9) | 1089 (45.8) | 263 (59.1) |
| Doses of COVID-19 Vaccine, n (%) |  |  |  |
| 0 | 2410 (85.9) | 2040 (86.4) | 370 (83.1) |
| 1 | 119 (4.2) | 82 (3.5) | 37 (8.3) |
| 2 | 231 (8.2) | 202 (8.6) | 29 (6.5) |
| 3 | 46 (1.6) | 37 (1.6) | 9 (2) |
| Death, n (%) | 520 (18.4) | 432 (18.2) | 88 (19.8) |

Supplementary Table 1. Characteristics of the study population according to being tested or not for SARS-CoV-2 viremia at hospital admission.

List of abbreviations: n, number; IQR, Inter Quartile Range; CCI, Charlson comorbidity index.
