# Supplementary material for: SARS-CoV-2 viremia and COVID-19 mortality: A prospective observational study
Source: PLoS One. 2023 Apr 28;18(4):e0281052. doi: 10.1371/journal.pone.0281052 (PMC10146509; doi:10.1371/journal.pone.0281052)
Supplement: S2 Table — (DOCX) [file pone.0281052.s005.docx]

| Characteristic | Overall  445 (100%) | Negative  SARS-CoV-2 viremia  359  (80.7%) | Positive  SARS-CoV-2 viremia  86  (19.3%) |
| --- | --- | --- | --- |
| Male biological sex, n (%) | 289 (64.9) | 231 (64.3) | 58 (67.4) |
| Age, years |  |  |  |
| median (IQR) | 65 (55-75) | 65 (56-749 | 67 (55-76) |
| >75 years, n (%) | 120 (27) | 94 (26.2) | 26 (32) |
| CCI, median (IQR) | 2.5 (1-4) | 3 (1-4) | 2 (1-5) |
| SARS-CoV-2 pandemic wave, n (%) |  |  |  |
| 1 | 32 (7.2) | 1 (0.3) | 31 (35.6) |
| 2 | 47 (10.5) | 43 (12) | 4 (4.7) |
| 3 | 255 (57.3) | 217 (60.4) | 38 (44.2) |
| 4 | 111 (24.9) | 98 (27.3) | 13 (15.1) |
| Days from symptoms onset to Hospital admission, median (IQR) | 8 (5-11) | 8 (5-11) | 7 (5-11) |
| Disease severity at hospital admission, n (%) |  |  |  |
| Mild/moderate | 182 (40.9) | 154 (43) | 28 (31.5) |
| Severe/critical | 263 (59.1) | 205 (57.1) | 58 (67.4) |
| Doses of COVID-19 Vaccine, n (%) |  |  |  |
| 0 | 370 (83.1) | 291 (81.1) | 79 (91.9) |
| 1 | 37 (8.3) | 34 (9.5) | 3 (3.5) |
| 2 | 29 (6.5) | 26 (7.2) | 3 (3.5) |
| 3 | 9 (2) | 8 (2.2) | 1 (1.2) |
| Death, n (%) | 88 (19.8) | 58 (16.2) | 30 (34.9) |

Supplementary Table 2. Characteristics of the study population according to being tested positive or negative for SARS-CoV-2 viremia.

List of abbreviations: n, number; IQR, Inter Quartile Range; CCI, Charlson comorbidity index.
