# Supplementary material for: SARS-CoV-2 viremia and COVID-19 mortality: A prospective observational study
Source: PLoS One. 2023 Apr 28;18(4):e0281052. doi: 10.1371/journal.pone.0281052 (PMC10146509; doi:10.1371/journal.pone.0281052)
Supplement: S3 Table — (DOCX) [file pone.0281052.s006.docx]

| Characteristics | Overall  1346  (100%) | Not tested for SARS-CoV-2 viremia  982  (72.9%) | Tested for SARS-CoV-2 viremia  364  (27.1%) |
| --- | --- | --- | --- |
| Male biological sex, n (%) | 802 (59.6) | 575 (58.6) | 227 (62.4) |
| Age, years |  |  |  |
| median (IQR) | 66 (55-78) | 66 (54-78) | 66 (56-75) |
| >75 years, n (%) | 446 (33.1) | 343 (34.9) | 103 (28.3) |
| CCI, median (IQR) | 3 (1-5) | 3 (1-5) | 3 (1-4) |
| SARS-CoV-2 pandemic wave, n (%) |  |  |  |
| 3 | 742 (55.1) | 487 (49.6) | 255 (70.1) |
| 4 | 604 (44.9) | 495 (50.4) | 109 (29.9) |
| Days from symptoms onset to Hospital admission, median (IQR) | 7 (5-10) | 7 (4-10) | 8 (5-11) |
| Disease severity at hospital admission, n (%) |  |  |  |
| Mild/moderate | 711 (52.8) | 560 (57) | 151 (41.5) |
| Severe/critical | 635 (47.2) | 422 (43) | 213 (58.5) |
| Doses of COVID-19 Vaccine, n (%) |  |  |  |
| 0 | 989 (74.3) | 699 (72.3) | 290 (79.7) |
| 1 | 111 (8.3) | 74 (7.7) | 37 (10.2) |
| 2 | 189 (14.2) | 161 (16.6) | 28 (7.7) |
| 3 | 42 (3.2) | 33 (3.4) | 9 (2.5) |
| Death, n (%) | 189 (14) | 119 (12.1) | 70 (19.2) |

Supplementary Table 3. Characteristics of the study population according to being tested or not for SARS-CoV-2 viremia at hospital admission restricted to the 3^rd^ and 4^th^ epidemic waves.

List of abbreviations: n, number; IQR, Inter Quartile Range; CCI, Charlson comorbidity index.
