# Supplementary material for: SARS-CoV-2 viremia and COVID-19 mortality: A prospective observational study
Source: PLoS One. 2023 Apr 28;18(4):e0281052. doi: 10.1371/journal.pone.0281052 (PMC10146509; doi:10.1371/journal.pone.0281052)
Supplement: S4 Table — (DOCX) [file pone.0281052.s007.docx]

| Characteristic | Overall  364  (100%) | Alive  294  (80.8%) | Death  70  (19.2%) |
| --- | --- | --- | --- |
| Male biological sex, n (%) | 227 | 183 | 44 |
| Age, years |  |  |  |
| median (IQR) | 66 (56-75) | 63 (54-72) | 76 (68-83) |
| >75 years, n (%) | 103 (28.3) | 62 (21.1) | 41 (58.6) |
| CCI, median (IQR) | 3 (1-4) | 2 (1-4) | 4 (3-6) |
| SARS-CoV-2 pandemic wave, n (%) |  |  |  |
| 3 | 255 (70.1) | 200 (68) | 55 (78.6) |
| 4 | 109 (29.9) | 94 (32) | 15 (21.4) |
| Days from symptoms onset to Hospital admission, median (IQR) | 8 (5-11) | 8 (5-11) | 7 (4-11) |
| Disease severity at hospital admission, n (%) |  |  |  |
| Mild/moderate | 150 (41.2) | 131 (44.6) | 20 (28.6) |
| Severe/critical | 213 (58.5) | 163 (55.4) | 50 (71.4) |
| Doses of COVID-19 Vaccine, n (%) |  |  |  |
| 0 | 290 (79.7) | 234 (79.6) | 56 (80) |
| 1 | 37 (10.2) | 32 (10.9) | 5 (7.1) |
| 2 | 28 (7.7) | 21 (7.1) | 7 (10) |
| 3 | 9 (2.5) | 7 (2.4) | 2 (2.9) |
| Positive SARS-CoV-2 viremia at admission, n (%) | 90 (24.7) | 52 (17.7) | 38 (54.3) |

Supplementary Table 4. Characteristics of the study population according to being alive or death after hospitalization for COVID-19 restricted to the 3^rd^ and 4^th^ epidemic waves.

List of abbreviations: n, number; IQR, Inter Quartile Range; CCI, Charlson comorbidity index.
