# Supplementary material for: SARS-CoV-2 viremia and COVID-19 mortality: A prospective observational study
Source: PLoS One. 2023 Apr 28;18(4):e0281052. doi: 10.1371/journal.pone.0281052 (PMC10146509; doi:10.1371/journal.pone.0281052)
Supplement: S5 Table — (DOCX) [file pone.0281052.s008.docx]

| Characteristic | Overall  364  (100%) | Negative  SARS-CoV-2 viremia  314  (86.3%) | Positive  SARS-CoV-2 viremia  50  (13.7%) |
| --- | --- | --- | --- |
| Male biological sex, n (%) | 227 (62.4) | 198 (63.1) | 29 (58) |
| Age, years |  |  |  |
| median (IQR) | 66 (56-75) | 65 (56-74) | 72 (61-78) |
| >75 years, n (%) | 103 (28.3) | 83 (26.4) | 20 (40) |
| CCI, median (IQR) | 3 (1-4) | 3 (1-4) | 3 (2-5) |
| SARS-CoV-2 pandemic wave, n (%) |  |  |  |
| 3 | 255 (70.1) | 217 (69.1) | 38 (76) |
| 4 | 109 (29.9) | 97 (30.9) | 12 (24) |
| Days from symptoms onset to Hospital admission, median (IQR) | 8 (5-11) | 8 (5-11) | 7 (4-10) |
| Disease severity at hospital admission, n (%) |  |  |  |
| Mild/moderate | 150 (41.2) | 137 (43.6) | 14 (28) |
| Severe/critical | 213 (58.5) | 177 (56.4) | 36 (72) |
| Doses of COVID-19 Vaccine, n (%) |  |  |  |
| 0 | 290 (79.7) | 246 (78.3) | 44 (88) |
| 1 | 37 (10.2) | 34 (10.8) | 3 (6) |
| 2 | 28 (7.7) | 26 (8.3) | 2 (4) |
| 3 | 9 (2.5) | 8 (2.5) | 1 (2) |
| Death, n (%) | 70 (19.2) | 51 (16.2) | 19 (38) |

Supplementary Table 5. Characteristics of the study population according to being tested positive or negative for SARS-CoV-2 viremia restricted to the 3^rd^ and 4^th^ epidemic waves.

List of abbreviations: n, number; IQR, Inter Quartile Range; CCI, Charlson comorbidity index.
